# Erythropoietin and iron for anemia in HIV-infected patients undergoing maintenance hemodialysis in China: a cross-sectional study

**DOI:** 10.1186/s12882-022-02693-y

**Published:** 2022-02-08

**Authors:** Lei Peng, Yanan He, Jiong Zhang, Daqing Hong, Guisen Li

**Affiliations:** 1 Department of Nephrology, Sichuan Provincial People’s Hospital, University of Electronic Science and Technology of China, Chengdu, 611731 China; 2 School of Automation Engineering, University of Electronic Science and Technology of China, Chengdu, 611731 China; 3 Public Health Clinical Center of Chengdu, Chengdu, China

**Keywords:** Erythropoietin, Iron, Anemia, HIV, Hemodialysis

## Abstract

**Background:**

Anemia is a common complication of chronic kidney disease (CKD) and HIV infection. The number of people living with HIV on hemodialysis (HD) is increasing. However, there is no data about anemia and related therapies in this kind of patients in China. We aim to assess the difference in hemoglobin (Hgb) and treatments like erythropoietin and iron between HIV-HD patients and HD patients in Chengdu, China.

**Methods:**

This cross-sectional study was conducted with data collection from January 2020 to June 2020. Thirty-four HIV-infected HD patients and thirty-five non-HIV-infected HD patients were included. Age, gender, dialysis vintage, single-pool (sp) Kt/V, Hgb, the dose of erythropoietin, ferritin, use of iron preparations, and serum albumin were collected in all patients. Time since HIV diagnosis, counts of CD4 + T cells, HIV RNA, and antiretroviral therapy for HIV infection were collected in HIV-infected patients. T-test, Mann–Whitney U test, and chi-square statistics were applied in SPSS.

**Results:**

The Hgb of HIV-HD and HD groups were 105.70 (95.93–112.08) g/L and 112.00 (93.00–126.00) g/L respectively (*P* = 0.064). There was a statistically significant higher erythropoietin dosage used in the HIV-HD population (222.55 ± 115.47 U/kg/week) compared to the HIV-negative HD group (161.86 ± 110.31 U/kg/week) (*P* = 0.029). 16/34 (47.06%) HIV-HD patients and 5/35 (14.29%) HD patients were treated with iron preparations (*P* = 0.003). The ferritin levels were 316.50 (117.38–589.75) ng/ml and 272.70 (205.00–434.00) ng/ml in HIV-HD and HD groups respectively.

**Conclusions:**

A higher erythropoietin dosage and a higher probability of iron preparations may be required to maintain Hgb in HIV-HD patients compared with HD patients.

**Supplementary Information:**

The online version contains supplementary material available at 10.1186/s12882-022-02693-y.

## Background

The incidences of acquired immunodeficiency syndrome (AIDS) associated events and death among people living with HIV (PLWH) have reduced over the last two decades due to increasing HIV testing and antiretroviral therapy (ART) [1]. However, non-AIDS-defining diseases (NADs), such as cardiovascular disease and kidney disease, were more prevalent in PLWH than HIV-uninfected individuals, which have become the main causes of death among PLWH in the era of modern ART [2, 3]. In a cohort of PLWH in China during 2010–2019, the incidence of advanced kidney disease was 270 per 100,000 person-years of follow-up after initiation of ART, which has become the second common NADs [4]. ART, infections (including HIV, BK virus, et.al), HIV-associated nephropathy, HIV-immune complex disease, and HIV-thrombotic thrombocytopenic purpura / hemolytic uremic syndrome may result in renal lesions and may eventually progress to end-stage renal disease (ESRD) which need dialysis treatment [5–7]. The number of PLWH on dialysis is increasing and survival rates of these patients are similar to those in the HIV-negative dialysis population [5, 8]. However, in another clinical trial, the one-year survival of HIV-positive hemodialysis (HD) patients seems to be lower compared to their HIV-negative counterparts [9] and the hemoglobin (Hgb) of HIV-positive dialysis patients was lower than that of HIV-negative people [10].

Anemia is a common complication of chronic kidney disease (CKD) and HIV infection [11, 12] and is associated with an increased risk of cardiovascular events, and all-cause mortality [13]. Thus, correction of anemia is an important task in the management of CKD complications. There are many reasons for anemia in HIV-HD patients. HIV infection may cause defective myelopoiesis/erythropoiesis, as well as the accumulation of myeloid/erythroid precursors, and may increase levels of inflammatory cytokines, affecting dynamics and functions or inducing Fas-mediated apoptosis of hematopoietic stem/progenitor cells, which consist of progenitors for all blood cell lineages including erythroid progenitors [14]. Opportunistic infections, antiretroviral drugs, other drugs administered to treat infection, and malignancies in HIV-positive patients could exacerbate the severity of anemia [15]. Relative erythropoietin deficiency, shortened erythrocyte lifespan, increased blood loss, chronic inflammation, iron deficiency, copper deficiency, vitamin B12 deficiency, folate deficiency, and aluminum overload result in anemia in HD patients [12]. Therefore, anemia in HIV-HD patients is theoretically more difficult to control. In fact, the Hgb of HIV-positive dialysis patients was lower than that of HIV-negative patients in a previous clinical trial [10]. Therapy with erythropoiesis-stimulating agents (ESAs) is an appropriate treatment for anemia in HIV-HD patients. Iron may be important in the activation of HIV-1 and high iron stores may also adversely influence the outcome of HIV-infected patients. Closely monitoring HIV disease progression in patients receiving intravenous iron by measuring plasma viral loads and CD4 + T cell counts is essential. Blood transfusions in HIV-infected patients with CKD should be avoided as some studies have shown increased activation of HIV-1 after transfusion, which could potentially accelerate HIV disease.[15]. The response to erythropoietin in HIV-infected patients appears to be similar to HIV-negative patients in a small study of HIV-HD patients [16]. However, in another study, there was a statistically significant higher erythropoietin dosage used in the HIV-HD patients as compared to the control group [17]. Up to now, there is no research on anemia and its treatment in HD patients with HIV infection in China. This study focused on anemia and its therapeutic strategy in southwestern areas of China and further explored the current status of anemia in HIV-HD patients and the optimization of treatment options.

## Materials and methods

### Patients and data collection

We conducted this cross-sectional study with data collection from January 2020 to June 2020. Thirty-four HIV-infected adult patients from the Public Health Clinical Center of Chengdu were enrolled. A group of HIV-negative patients were then selected as control in a 1:1 ratio from Sichuan Provincial People's Hospital in China. Available influencing factors on Hgb including age, gender, dialysis vintage, single-pool (sp) Kt/V, serum albumin, frequency of hospitalizations, frequency of infection and medications were compared and considered when selecting the control group. Thirty-five non-HIV- infected patients were selected from 394 patients in the study. All participating patients aged 18 to 75 years were on maintenance HD therapy ≥ 3 months and received HD three times a week. The diagnosis of HIV/AIDS was based on the Diagnostic Criteria for HIV/AIDS (WS 293–2008) [18]. The patients who had hematologic diseases causing anemia (including aplastic anemia, hemolytic anemia, bleeding, and so on), severe active infection, and tumors were excluded. Erythropoietin which enrolled patients used were all of domestic origin.

Age, gender, dialysis vintage, single-pool (sp) Kt/V, serum albumin, frequency of hospitalizations, frequency of infection, medications, Hgb, the dose of erythropoietin, ferritin and use of iron preparations were collected in all patients from January 2020 to June 2020. Time since HIV diagnosis, counts of CD4 + T cells, HIV RNA, and antiretroviral therapy for HIV infection were collected in HIV-infected patients. Multiple data in different time points of one parameter in the same patient were expressed as mean. This study was approved by the ethics committee of Sichuan Provincial People's Hospital (No. 2017–124). All participants provided written informed consent. All procedures in this study were in accordance with the Declaration of Helsinki.

### Statistical analysis

The normality of continuous data was evaluated using the Shapiro–Wilk test. Normally distributed data were expressed as means ± standard deviations (SDs) and assessed using the t-test, whereas non-normally distributed data were expressed as median and interquartile range (IQR) and the Mann Whitney U test was applied. The chi-square statistics or Fisher’s exact test were used for categorical variables. Hgb, ferritin, dialysis vintage, hospitalizations, infections and serum albumin were compared using the Mann Whitney U test. Weekly erythropoietin dose, age and spKt/V were compared using the t-test. Gender, medication and proportion of treatment with iron preparations were compared using chi-square statistics or Fisher’s exact test. Statistical analyses were conducted in SPSS (version 26.0). A value of *p* < 0.05 was considered significant.

## Results

### Baseline characteristics of enrolled patients

Thirty-four HIV-infected patients and thirty-five non-HIV-infected patients were included in the study with data collection from January 2020 to June 2020. The baseline characteristics are presented in Table 1. Gender, age, dialysis vintage, spKt/V, serum albumin, hospitalizations, infections and medications of HIV group are not statistically different from the control group except the proportion of treatment with α-ketoacid. The different proportion in α-ketoacid usage may be due to different health coverage policy in two dialysis centers. However, serum albumin of the two groups showed no statistically difference indicating that this disparity had no impact on the nutritional status. The two groups were well matched.

**Table 1:** Baseline characteristics of enrolled patients

Group	Control (*n* = 35)	HIV (*n* = 34)
Gender (male: female)	29: 6	29: 5
Age (year)	44.69 ± 8.88	45.18 ± 7.73
Dialysis vintage (month)	25 (17–37)	26 (13–36)
spKt/V	1.34 ± 0.23	1.27 ± 0.16
Serum albumin (g/L)	42.10 (40.35–43.40)	41.95 (38.88–43.55)
Hospitalizations (times/6 m)	0 (0–0)	0 (0–1)
Infections (times/6 m)	0 (0–0)	0 (0–0)
Medications		
Antihypertensive drugs (Y/N)	33/2	31/3
Phosphate binders (Y/N)	34/1	31/3
Active vitamin D or Cinacalcet (Y/N)	33/2	34/0
α-Ketoacid (Y/N)	13/22	2/32^*^

Dialysis vintage, serum albumin, hospitalizations and infections expressed as median *IQR*, Age and spKt/V expressed as means ±SEM. Results of HIV group are not statistically different from control group except the proportion of treatment with α-ketoacid.

*p < 0.05 compared to control.

### Clinical characteristics of HIV infected patients

Thirty-four HIV-infected patients were enrolled in this study. The mean time since the HIV diagnosis was 49.62 ± 28.86 months. Except for two patients for personal reasons, antiretroviral drugs were administrated in the remaining 32 patients. Five HIV patients had no results of CD4 + T cell counts from January 2020 to June 2020. Of the remaining 29 HIV patients, 7 (24.1%) patients had a CD4 + T cell count of fewer than 200 cells/mm^3^, and 5 (17.2%) patients had a CD4 + T cell count of more than 500 cells/mm^3^. Twelve HIV patients had no results of HIV-RNA from January 2020 to June 2020. Of the remaining 22 HIV patients, only 1 (4.5%) patient had an HIV viral load above the lower detection limit. The possible reason was the short time of diagnosis and treatment for this patient (Time since HIV diagnosis: 4 months). The above data in HIV-HD group indicated relatively stable state of HIV infection in these patients.

### Hgb and dose of erythropoietin

The Hgb and responses to erythropoietin in HIV-infected HD patients and HD patients are shown in Table 2. The Hgb of HIV-HD and HD groups were 105.70 (95.93–112.08) g/L and 112.00 (93.00–126.00) g/L respectively. Although there was a trend toward higher hgb levels in the control population, it did not reach statistical significance (*P* = 0.064). 4/34 (11.76%) patients in the HIV-HD group had Hgb < 90 g/L, compared with 2/35 (5.71%) in the HD group (*P* = 0.642). There was a statistically significant higher erythropoietin dosage used in the HIV population (222.55 ± 115.47 U/kg/week) compared to the HIV-negative group (161.86 ± 110.31 U/kg/week) (*P* = 0.029).

**Table 2:** Hemoglobin and dose of erythropoietin in HIV-infected patients on hemodialysis

Group	Control (*n* = 35)	HIV (*n* = 34)
Hemoglobin (g/L)	112.00 (93.00–126.00)	105.70 (95.93–112.08)
Weekly erythropoietin dose (U/kg)	161.86 ± 110.31	222.55 ± 115.47^*^

Hemoglobin expressed as median (IQR); Weekly erythropoietin expressed as means ± SEM

^*^p < 0.05 compared to control

### Ferritin and treatment with iron preparations

The proportion of treatment with iron preparations and ferritin in HIV-infected HD patients and control HD patients are presented in Table 3. 16/34 (47.06%) HIV-HD patients and 5/35 (14.29%) HD patients were treated with iron preparations (*P* = 0.003). The details of iron administration showed in supplemental table. The ferritin levels were 316.50 (117.38–589.75) ng/ml and 272.70 (205.00–434.00) ng/ml in HIV-HD and HD groups respectively (*P* = 0.653).

**Table 3:** Ferritin and treatment with iron preparations

Group	Control (*n* = 35)	HIV (*n* = 34)
Ferritin (ng/ml)	272.70 (205.00–434.00)	316.50 (117.38–589.75)
Proportion of treatment with iron preparations	5/35	16/34^*^

Ferritin expressed as median (IQR)

^*^p < 0.05 compared to control

## Discussion

For adult HD patients, KDIGO suggests that ESAs therapy is used to avoid having the Hgb concentration fall below 90 g/L by starting erythropoietin therapy when the Hb is between 90–100 g/L in 2012 [19]. Our study showed that 11.76% of patients in the HIV-HD group and 5.71% of patients in the HD group had Hgb < 90 g/L, compared with 18.8% patients in China Dialysis Outcomes and Practice Patterns Study (DOPPS) [20]. The reason for the lower prevalence of Hgb < 90 g/L maybe include younger age, shorter dialysis vintage, higher percentage of males and higher dialysis frequency in enrolled patients in our study (Table 4) [20]. There was no significant difference in Hgb and ferritin between HIV-HD and HD patients in our study. However, higher ESAs dosage and higher rate of treatment with iron preparations were observed in HIV-HD patients compared with controlled HD patients. Patients in our study used oral and intravenous iron and various effectiveness of different iron dosage forms made it difficult to compared the iron dose of the two groups quantitatively. Therefore, we compared the rates of treatment with iron preparations in two groups only.

**Table 4:** Different baseline characteristics of this study and DOPPS

Group	Control (*n* = 35)	HIV (*n* = 34)	China subgroup of DOPPS (*n* = 1427)
Gender (male%)	82.9	85.3	54.6
Age (year)	44.69 ± 8.88	45.18 ± 7.73	58.7 ± 3.5
Dialysis vintage (month)	25.0 (17.0–37.0)	26.0 (13.0–36.0)	40.8 (18.0–75.6)
Dialysis frequency			
3-times per week (%)	100	100	79.8
2-times per week (%)	0	0	20.2

*DOPPS*, Dialysis Outcomes and Practice Patterns Study. Dialysis vintage expressed as median *IQR*, Age expressed as means ±SEM

The reasons for anemia in HIV-HD patients are complex. Increased levels of inflammatory cytokines, defective erythropoiesis, opportunistic infections, antiretroviral drugs, other drugs administered to treat infection, and malignancies in HIV-positive patients could aggravate the severity of anemia [14, 15]. Among all the reasons, systemic inflammation suppresses Hgb response to ESAs [21]. Therefore, we suppose that inflammation caused by HIV infection may reduce the sensibility to ESAs and lead to an increase in the demand for ESAs in HIV-HD patients. Unfortunately, the patients in this study did not routinely detect C-reactive protein and other inflammatory indicators, which made it impossible to assess the inflammatory state. More ESAs means more adverse effect including hypertension and thrombotic events, et al. [22]. Several studies have shown that severe ESA hyporesponsiveness is associated with poor survival and suggested that even higher ESA doses might contribute to poor patient outcomes [23].

There are limited therapies for anemia in HIV-HD patients. Classical treatment includes ESAs, iron therapy, and blood transfusions. Higher dosage of ESAs and higher proportion of iron use were observed in this study in HIV-HD patients which may be attributed to the poor ESA responsiveness caused by HIV infection and may furtherly lead to more side effects and poor outcomes. However, apparent inflammation did not appear to affect the Hgb response with roxadustat [24], a hypoxia-inducible factor (HIF) prolyl hydroxylase inhibitor. HIF prolyl hydroxylase inhibitor like roxadustat and vadadustat which has been used for CKD–related anemia in recent years are oral and being developed to stabilize HIF and thereby mimic a state of cellular hypoxia [24, 25]. HIF prolyl hydroxylase inhibitor might be a new choice for anemia in HIV-HD patients.

## Conclusion

In HIV-HD patients, a higher erythropoietin dosage and a higher proportion of iron use were observed compared with HD patients, however, there were no significant differences in Hgb and ferritin between the two groups. We supposed that inflammation caused by HIV infection maybe the reason of ESA hyporesponsiveness in HIV-HD patients. Further studies are required to confirm this hypothesis.

## Supplementary Information

**Additional file 1:** **Table.** Administration ofiron in the enrolled patients.

## Data Availability

Additional data and materials are available in REDCap.samsph.com:16,866 on reasonable request.

## Abbreviations

CKD: Chronic Kidney Disease
HD: Hemodialysis
Hgb: Hemoglobin
AIDS: Acquired immunodeficiency syndrome
PLWH: People living with HIV
ART: Antiretroviral therapy
NADs: Non-AIDS-defining diseases
ESRD: End-stage renal disease
ESAs: Erythropoiesis-stimulating agents
SDs: Standard deviations
