# Supplementary material for: Erythropoietin and iron for anemia in HIV-infected patients undergoing maintenance hemodialysis in China: a cross-sectional study
Source: BMC Nephrol. 2022 Feb 8;23:60. doi: 10.1186/s12882-022-02693-y (PMC8827246; doi:10.1186/s12882-022-02693-y)
Supplement: Supplementary file 1 — Additional file 1: Table. Administration ofiron in the enrolled patients. [file 12882_2022_2693_MOESM1_ESM.docx]

| Patient  Table. Administration of iron in the enrolled patients. | Usage of iron (Y/N) | Dosage  forms (Oral/IV) | Dose of elemental iron (mg) | | | | | |
| --- | --- | --- | --- | --- | --- | --- | --- | --- |
|  |  |  | Jan | Feb | Mar | Apr | May | Jun |
| HIV1 | Y | Oral | 4500 | 4500 | 4500 | 4500 | 4500 | 4500 |
| HIV2 | Y | IV | 800 | 800 | 800 | 800 | 800 | 800 |
| HIV3 | N | - | - | - | - | - | - | - |
| HIV4 | N | - | - | - | - | - | - | - |
| HIV5 | Y | Oral | 4500 | 4500 | 4500 | 4500 | 4500 | 4500 |
| HIV6 | N | - | - | - | - | - | - | - |
| HIV7 | N | - | - | - | - | - | - | - |
| HIV8 | Y | Oral | 4500 | 4500 | 4500 | 4500 | 4500 | 4500 |
| HIV9 | Y | Oral | 4500 | 4500 | 4500 | 4500 | 4500 | 4500 |
| HIV10 | Y | Oral | 0 | 0 | 4500 | 4500 | 4500 | 4500 |
| HIV11 | N | - | - | - | - | - | - | - |
| HIV12 | N | - | - | - | - | - | - | - |
| HIV13 | N | - | - | - | - | - | - | - |
| HIV14 | N | - | - | - | - | - | - | - |
| HIV15 | Y | Oral | 4500 | 4500 | 4500 | 4500 | 4500 | 4500 |
| HIV16 | N | - | - | - | - | - | - | - |
| HIV17 | N | - | - | - | - | - | - | - |
| HIV18 | Y | Oral | 0 | 4500 | 4500 | 4500 | 4500 | 4500 |
| HIV19 | N | - | - | - | - | - | - | - |
| HIV20 | N | - | - | - | - | - | - | - |
| HIV21 | N | - | - | - | - | - | - | - |
| HIV22 | Y | Oral | 4500 | 4500 | 4500 | 4500 | 4500 | 4500 |
| HIV23 | Y | Oral | 4500 | 4500 | 4500 | 4500 | 4500 | 4500 |
| HIV24 | N | - | - | - | - | - | - | - |
| HIV25 | Y | Oral | 4500 | 4500 | 4500 | 0 | 0 | 0 |
| HIV26 | N | - | - | - | - | - | - | - |
| HIV27 | N | - | - | - | - | - | - | - |
| HIV28 | N | - | - | - | - | - | - | - |
| HIV29 | Y | Oral | 4500 | 4500 | 4500 | 4500 | 4500 | 4500 |
| HIV30 | Y | Oral | 4500 | 4500 | 4500 | 4500 | 4500 | 4500 |
| HIV31 | N | - | - | - | - | - | - | - |
| HIV32 | Y | Oral | 4500 | 4500 | 4500 | 4500 | 4500 | 4500 |
| HIV33 | Y | IV | 0 | 0 | 800 | 800 | 800 | 0 |
| HIV34 | Y | Oral | 4500 | 4500 | 4500 | 4500 | 4500 | 4500 |
| CON1 | N | - | - | - | - | - | - | - |
| CON2 | N | - | - | - | - | - | - | - |
| CON3 | N | - | - | - | - | - | - | - |
| CON4 | Y | IV | 800 | 800 | 800 | 800 | 800 | 800 |
| CON5 | N | - | - | - | - | - | - | - |
| CON6 | N | - | - | - | - | - | - | - |
| CON7 | N | - | - | - | - | - | - | - |
| CON8 | Y | IV | 800 | 400 | 0 | 0 | 0 | 0 |
| CON9 | N | - | - | - | - | - | - | - |
| CON10 | N | - | - | - | - | - | - | - |
| CON11 | N | - | - | - | - | - | - | - |
| CON12 | N | - | - | - | - | - | - | - |
| CON13 | N | - | - | - | - | - | - | - |
| CON14 | N | - | - | - | - | - | - | - |
| CON15 | N | - | - | - | - | - | - | - |
| CON16 | N | - | - | - | - | - | - | - |
| CON17 | N | - | - | - | - | - | - | - |
| CON18 | N | - | - | - | - | - | - | - |
| CON19 | N | - | - | - | - | - | - | - |
| CON20 | N | - | - | - | - | - | - | - |
| CON21 | N | - | - | - | - | - | - | - |
| CON22 | N | - | - | - | - | - | - | - |
| CON23 | Y | IV | 0 | 1000 | 200 | 0 | 0 | 0 |
| CON24 | Y | IV | 1000 | 200 | 0 | 0 | 0 | 0 |
| CON25 | N | - | - | - | - | - | - | - |
| CON26 | N | - | - | - | - | - | - | - |
| CON27 | N | - | - | - | - | - | - | - |
| CON28 | N | - | - | - | - | - | - | - |
| CON29 | N | - | - | - | - | - | - | - |
| CON30 | N | - | - | - | - | - | - | - |
| CON31 | Y | IV | 1000 | 1000 | 1000 | 1000 | 1000 | 400 |
| CON32 | N | - | - | - | - | - | - | - |
| CON33 | N | - | - | - | - | - | - | - |
| CON34 | N | - | - | - | - | - | - | - |
| CON35 | N | - | - | - | - | - | - | - |

HIV: HD patients with HIV; CON: HD patients without HIV; IV: Intravenous;
